# The Impact of Age and Pathogens Type on the Gut Microbiota in Infants with Diarrhea in Dalian, China

**DOI:** 10.1155/2020/8837156

**Published:** 2020-11-30

**Authors:** Qingjie Fan, Ming Yi, He Liu, Yushuang Wang, Xinke Li, Jieli Yuan, Lili Wang, Binbin Hou, Ming Li

**Affiliations:** ^1^College of Basic Medical Science, Dalian Medical University, Dalian, China; ^2^Center for Disease Control (CDC) of Xigang District, Dalian, China; ^3^The Second Hospital of Dalian Medical University, Dalian, China

## Abstract

**Objective:**

Diarrhea in infants is a serious gastrointestinal dysfunction characterized by vomiting and watery bowel movements. Without proper treatment, infants will develop a dangerous electrolyte imbalance. Diarrhea is accompanied by intestinal dysbiosis. This study compared the gut microbiota between healthy infants and diarrheic infants. It also investigated the effects of age and pathogen type on the gut microbiota of infants with diarrhea, providing data for the proper treatment for diarrhea in infants.

**Materials and Methods:**

DNA was collected from the fecal samples of 42 Chinese infants with diarrhea and 37 healthy infants. The healthy infants and infants with diarrhea were divided into four age groups: 0–120, 120–180, 180–270, and 270–365 days. Using PCR and 16S rRNA high-throughput sequencing, the diarrhea-causing pathogens in these infants were identified and then categorized into four groups: *Salmonella* infection, *Staphylococcus aureus* infection, combined *Salmonella* and *Staphylococcus aureus* infection, and others (neither *Salmonella* nor *Staphylococcus aureus*).

**Results:**

The species diversity of gut microbiota in diarrheic infants was significantly reduced compared with that in healthy infants. Infants with diarrhea had a lower abundance of *Lactobacillus* spp. and *Bacillus* spp. (*P* < 0.001) and a significant richness of *Klebsiella* spp. and *Enterobacter* spp. (*P* < 0.001). Similar gut microbiota patterns were found in diarrheic infants in all four age groups. However, different pathogenic infections have significant effects on the gut microbiota of diarrheic infants. For instance, the relative abundance of *Klebsiella* spp. and *Streptococcus* spp. was significantly increased (*P* < 0.001) in infants infected with *Staphylococcus aureus*; meanwhile, the richness of bacteria such as *Enterobacter* spp. was significantly increased in the *Salmonella* infection group (*P* < 0.001).

**Conclusion:**

The microbiota in infants with diarrhea has changed significantly, characterized by decreased species diversity and abundance of beneficial bacteria and significant increase in the proportion of conditional pathogens. Meanwhile, the gut microbiota of infants with diarrhea at different ages was similar, but different pathogenic infections affect the gut microbiota characteristics. Therefore, early identification of changes in gut microbiota in infants with diarrhea and the adoption of appropriate pathogen type-specific interventions may effectively alleviate the disease and reduce adverse reactions.

## 1. Introduction

As a serious gastrointestinal dysfunction, infant diarrhea has become a global public health problem. Without proper treatment, the child will have an electrolyte imbalance that can be life-threatening [1]. Diarrhea was the second cause of childhood mortality, according to the last WHO bulletin, published in 2018 (https://www.who.int/gho/publications/world_health_statistics/2018/en/). *Rotavirus*, *Shigella* spp., and *Salmonella* spp. were the three leading causes of diarrheal deaths in infants [2]. Also, *Staphylococcus aureus* was one of the common pathogens of infectious diarrhea [3]; the peptidoglycan and toxins of *S. aureus* can induce infantile diarrhea [4].

The gut microbiota community is symbiotic with the host and changes dynamically with the host's age and physiological status as well as environmental factors [5, 6]. The homeostasis of the intestinal microbiota plays a vital role in human health, specifically by promoting the digestion and absorption of food, maintaining the host's immune balance, metabolism, and homeostasis of the intestinal barrier [7, 8]. In addition, the emergence of probiotics, prebiotics, and other products provides new means of preventing and treating clinical diseases [9, 10].

However, once the host's intestinal microecological balance was broken, various intestinal diseases will follow [11]. For instance, infantile diarrhea was one of the most common metabolic diseases related to the infant's gut microecological balance. More research has found that gut dysbiosis has an impact on the occurrence and development of diarrhea. For example, The et al. have reported a consistent elevation of *Fusobacterium mortiferum*, *Escherichia*, and reduced *Bifidobacterium pseudocatenulatum* in infants with diarrhea [12]. Another research found *Bifidobacterium* and *Lactobacillus* species to be decreased in Colombian children with diarrhea [13]. However, few reports compared the gut microbiota characteristics in infants of different ages or with different pathogens.

This study aimed to (a) identify the differences in the gut microbiota composition between diarrheic (under one year) and healthy infants, (b) characterize the gut microbiota in diarrheic infants of different ages, and (c) examine the effect of different pathogenic bacteria on the intestinal microbiota of infants with diarrhea. This study can clarify gut microbiota changes in infants with diarrhea and provide a specific reference for the additional diagnosis and treatment.

## 2. Materials and Methods

### 2.1. Subjects and Sample Collection

**Figure 1:**
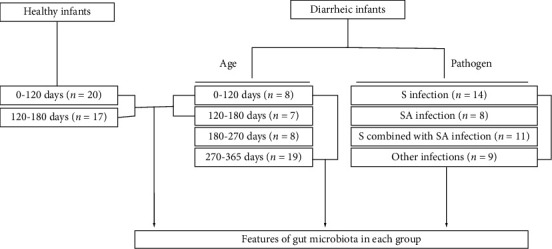
The flowchart of sample collection and grouping in this study. S: *Salmonella*; SA: *Staphylococcus aureus*.

A total of 42 diarrheic infants and 37 healthy infants under the age of one were recruited from the Center for Disease Control of Xigang District, Dalian, China (Figure 1, Table 1). Infants with virus infection, such as rotavirus, were excluded from the study; all infantile diarrhea cases were caused by prokaryotic infection. Meanwhile, infants with diarrhea were in the early stages of diarrhea and had not been treated with antibiotics. The fecal samples were collected from each infant and immediately stored at −20°C until transfer to the laboratory on dry ice and then stored at −80°C before use.

The study was approved by the ethical committees of Dalian Medical University, Dalian, China. Patients have filled out the informed consent form before sample collection.

### 2.2. Fecal DNA Extraction, PCR Amplification, 16S rRNA Sequencing, and Analysis

The microbial genomic DNA from the fecal samples was extracted using the E.Z.N.A.® Stool DNA kit (Omega Bio-tek, Inc.). The DNA concentration was measured using the Qubit 2.0 Fluorometer (Thermo Fisher Scientific, USA). PCR was performed to amplify the V3 and V4 region of the 16S rRNA gene using the primers 341F (5′-CCTAYGGGRBGCASCAG-3′) and 806R (5′-GGACTACNNGGGTATCTAAT-3′); template DNA was absent in the negative control [14]. PCR products were monitored on a 2% agarose gel. The PCR fragments were sequenced on an Illumina HiSeq platform (Novogene, Beijing, China). The QIIME software 1.9 package was used to analyze sequences (Quantitative Insights Into Microbial Ecology, http://bio.cug.edu.cn/qiime/). Sequences having a 97% resemblance or higher were categorized as the same operational taxonomic units (OTUs). The alpha diversity of microbiota was evaluated by the Chao 1 index, observed species index, and abundance-based coverage estimator (ACE) index. The beta diversity was evaluated by nonmetric multidimensional scaling (NMDS) [15]. The ANOSIM similarity analysis was based on a nonparametric test to compare intragroup and intergroup differences [16]. Linear discriminant analysis Effect Size (LEfSe) was used to identify the bacterial taxa differentially represented between groups at different taxonomic levels. A linear discriminant analysis (LDA) was used to estimate the effect size of each deferentially abundant feature (LDA ≥ 4 was shown in figures) [17]. The datasets are publicly available (accession number: PRJNA611095).

### 2.3. Identification of Different Pathogens in Feces of Diarrheic Infants

The PCR amplification of the partial 16S rRNA gene of *Salmonella* and *Staphylococcus aureus* was performed. The forward (5′-GTG AAA TTA TCG CCA CGT TCG GGC AA-3′) and reverse primer (5′-TCA TCG CA CCG TCA AAG GAA CC-3′) were used to detect a 284-bp *Salmonella* gene fragment [18, 19]. Notwithstanding, *Staphylococcus aureus* infection was identified by the PCR with the forward (5′-AAC TCT GTT ATT AGG GAA GAACA-3′) and reverse (5′-CCA CCT TCC GGT TTG TCA CC-3′ [20]) primer. This way, we divided the samples into four categories according to pathogen type: *Salmonella*, *Staphylococcus aureus*, combined *Salmonella* and *Staphylococcus aureus*, and others (neither *Salmonella* nor *Staphylococcus aureus*) (Table 1).

### 2.4. Statistical Analysis

All the experiments were done in triplicate. The data were presented as arithmetic mean ± standard error of the mean (SEM). Community comparisons were evaluated using a Student's *t*-test with the GraphPad Prism Program (Version 8.1.0; GraphPad Software Inc., La Jolla, CA, USA) [21]. The QIIME was used to calculate the beta diversity distance matrix, and the *R* language vegan software package was applied to perform NMDS analysis and mapping [22]. A *P* value of less than 0.05 was considered as statistically significant.

## 3. Results

### 3.1. Cohorts of Patients and Healthy Infants

The basic clinical information of 37 healthy infants and 42 diarrheic infants was collected, including gender, age, and number of samples in each category. 61.9% of infants with diarrhea have skin problems, such as pruritus and rash (Table 1). The healthy infants and infants with diarrhea were divided into four groups by age: 0–120, 120–180, 180–270, and 270–365 days. Using PCR and 16S rRNA sequencing, the diarrhea-causing pathogens in these infants were identified and divided into four categories, *Salmonella*, *Staphylococcus aureus*, combined *Salmonella* and *Staphylococcus aureus*, and others, which were neither *Salmonella* nor *Staphylococcus aureus* (Figure S1). To eliminate the effect of age on experimental results, we excluded 18 diarrheic infants to ensure no statistical difference in the arithmetic mean and SEM deviation of age between diarrheic and the healthy infants (*P*=0.897, Figure 1).

### 3.2. The Alterations of Gut Microbiota Composition in Diarrheic Infants

**Figure 2:**
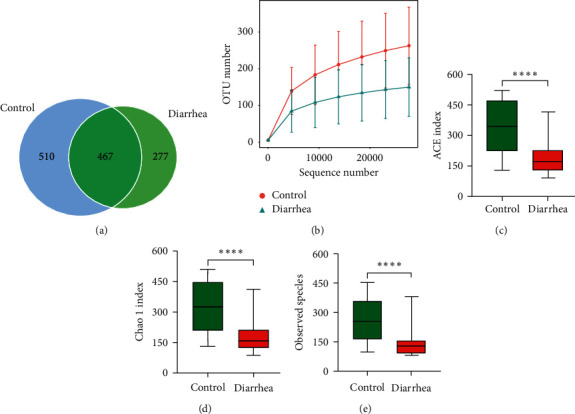
The alpha diversity of gut microbiota in healthy infants and diarrheic infants. (a) Venn diagram of OTUs in the two groups. (b) Observed species index in the two groups. (c–e) Comparing the alpha diversity indices (ACE, Chao 1, and observed species) based on the OTU profiles.

The overlapping OTUs of the healthy infant's group and the diarrhea group were shown in a Venn diagram (Figure 2(a)). The 16S rRNA gene sequencing showed 977 and 744 unique OTUs in healthy and diarrheic infants, respectively, while 467 OTUs were common in both groups. According to OTU analysis, the bacterial communities in diarrheic and healthy infants tended to be heterogeneous (Figure 2(b)). The ACE, Chao 1, and observed species index showed that gut microbiota of diarrheic infants had significantly lower alpha diversity than those of healthy infants (all *P*=0.001; Figures 2(c), 2(d) and 2(e)).

**Figure 3:**
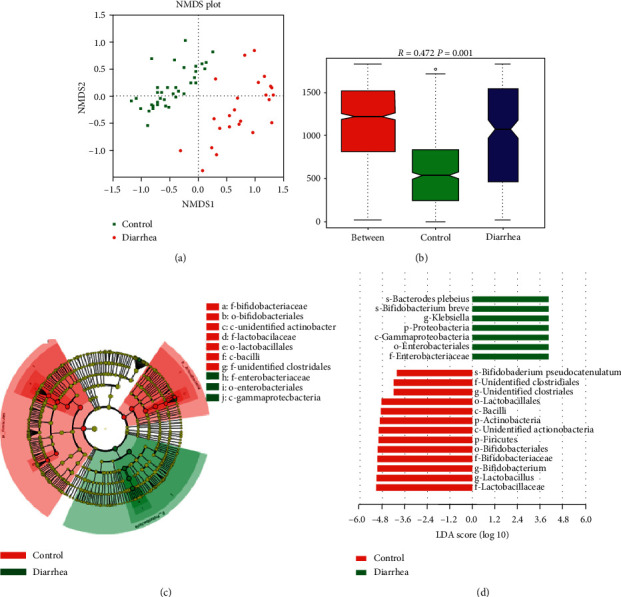
The beta diversity and predominant microbiota of healthy and diarrheic infants. (a) The nonmetric multidimensional scaling (NMDS) of beta diversity is calculated on ranking order. (b) The ANOSIM Similarity Analysis is based on a nonparametric test to compare intragroup and intergroup differences. (c) Cladogram indicating the phylogenetic distribution of microorganisms as related to group characteristics; the difference was shown in red for the healthy group and green for the diarrhea group. The diameter of each circle was proportional to the taxa's abundance. The strategy of multiclass analysis was not strict (at least one somewhat differential). The circle from inside to outside represented the phylogenetic level from domain to genus. (d) Indicator microbial groups within the two types of sediments with a linear discriminate analysis (LDA) value greater than 4.0. The color lump represented the microbes with a significant difference at different taxonomic levels. Red and green represented the healthy group and the diarrhea group, respectively. The *x*-axis represented the LDA score of the microbes. The *y*-axis represented the microbes, which were detected to be significantly different in the groups.

The NMDS calculation on ranking order was used for clustering the 79 samples into two distinct enterotypes (Figure 3(a)). The intergroup divergence was greater than intragroup divergence (Figure 3(b)), suggesting a significant difference in beta diversity between healthy infants and diarrheic infants. The LDA effect size (LefSe) algorithm was adopted to identify the bacterial groups that showed significant differences in abundance between the two groups. Comparisons between the two groups revealed that the *Firmicutes* phylum was significantly more abundant in healthy infants than diarrheic infants. At the genus level, the main abundant microbial genera shifted from *Lactobacillus* and *Bifidobacterium* in healthy infants to *Klebsiella* and *Streptococcus* in diarrheic infants (Figures 3(c) and 3(d)).

**Figure 4:**
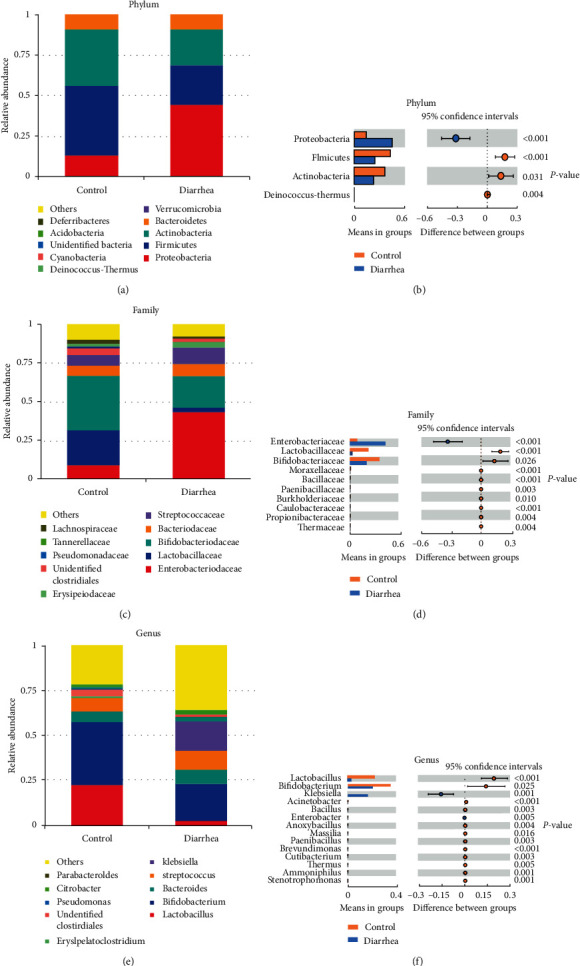
The shift in the gut microbiota of healthy infants and diarrheic infants. The relative abundance of the top 10 microbiota at the phylum (a), order (c), and genus level (e). The genera in the gut microbiota of healthy infants were strikingly different from those in diarrheic infants at the phylum (b), order (d), and genus level (f).

Distinct bacterial composition was observed between the healthy and diarrhea group. The microbiome contained 23 phyla, 168 families, and 370 genera in all fecal samples. Proteobacteria, Firmicutes, and Actinobacteria were the most abundant taxonomic groups. The relative abundance of Proteobacteria (44.67%) in diarrheic infants was substantially higher than healthy infants (*P* < 0.001), while the abundances of *Firmicutes* (24.27%) and *Actinobacteria* (22.14%) in diarrheic infants were lower (*P* < 0.001, *P*=0.031, Figures 4(a), and 4(b)). At the family level (Figures 4(c), 4(d)), the relative abundance of Enterobacteriaceae (43.60%) in diarrheic infants was considerably higher than in healthy infants (*P* < 0.001). On the contrary, the relative abundance of Lactobacillaceae (3.03%) and Bifidobacteriaceae (2.05%) in diarrheic infants was notably lower than in healthy infants (*P* < 0.001, *P*=0.026). At the genus level (Figures 4(e), 4(f)), the relative abundance of *Klebsiella* (16.57%) in diarrheic infants was higher than in healthy infants (*P*=0.001). On the contrary, the relative abundance of *Lactobacillus* (3.03%) and *Bifidobacterium* (20.52%) in diarrheic infants was lower than in healthy infants (*P* < 0.001, *P*=0.025).

### 3.3. Characteristics of Gut Microbiota in Healthy and Diarrheic Infants at Different Ages

**Figure 5:**
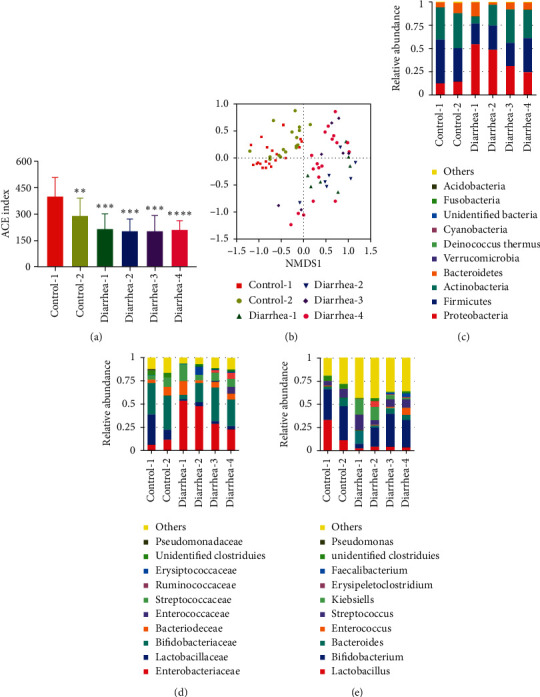
The gut microbiota of healthy infants and infants with diarrhea of different ages. (a). The comparison of the alpha diversity (ACE index) of various ages based on the OTUs profiles. (b). The NMDS of beta diversity is calculated on ranking order. The relative abundance of the top 10 microbiota at the phylum (c), order (d), and genus level (e). Control-1: 0–120-day-old healthy infants; control-2: 120–180-day-old healthy infants; diarrhea-1: 0–120-day-old diarrheic infants; diarrhea-2: 120–180-day-old diarrheic infants; diarrhea-3: 180–270-day-old diarrheic infants; diarrhea-4: 270–360-day-old diarrheic infants.

The healthy infants and infants with diarrhea were divided by age into four groups: 0–120, 120–180, 180–270, and 270–365 days. We investigated the characteristics of the gut microbiota of infants in the four age groups. The alpha diversity indexes showed that, in healthy infants, the diversity of gut microbiota of 120–180-day-old infants was significantly lower than that of 0–120-day-old infants (*P* < 0.01). Interestingly, different ages did not affect the diversity of the gut microbiota of diarrheic infants (Figure 5(a), *P* > 0.05). Based on the factor of age, NMDS clustering divided the samples of infants with diarrhea into four groups; however, the four groups did not separate clearly, suggested a similarity among the samples from different age groups (Figure 5(b)).

At the phylum level (Figure 5(c)), the relative abundance of Proteobacteria (54.67%) in diarrhea-1 (0–120 days) infants was individually higher than in diarrhea-4 (270–365 days) infants (*P*=0.027). On the contrary, the relative abundance of Actinobacteria (7.29%) in diarrhea-1 (0–120 days) infants was lower than in diarrhea-3 (180–270 days) infants (*P*=0.031) and diarrhea-4 (270–365 days) infants (*P*=0.013). At the family level (Figure 5(d)), the relative abundance of Enterobacteriaceae (53.96%) in diarrhea-1 infants was significantly higher than in diarrhea-4 infants (*P*=0.025). On the contrary, the relative abundance of Bifidobacteriaceae (5.07%) in diarrhea-1 infants was lower than in diarrhea-3 infants (*P*=0.024) and diarrhea-4 infants (*P*=0.009). At the genus level (Figure 5(e)), the relative abundance of *Bifidobacterium* (5.07%) in diarrhea-1 infants was lower than in diarrhea-3 infants (*P*=0.024) and diarrhea-4 infants (*P*=0.009). The relative abundance of *Klebsiella* (14.42%) in diarrhea-2 infants (120–180 days) was higher than in diarrhea-4 infants (*P*=0.031). Other than the previously mentioned comparisons, the differences among the other groups of diarrheic infants were not statistically significant.

### 3.4. The Characteristics of Intestinal Microbiota in Diarrheic Infants Infected with Different Pathogens

**Figure 6:**
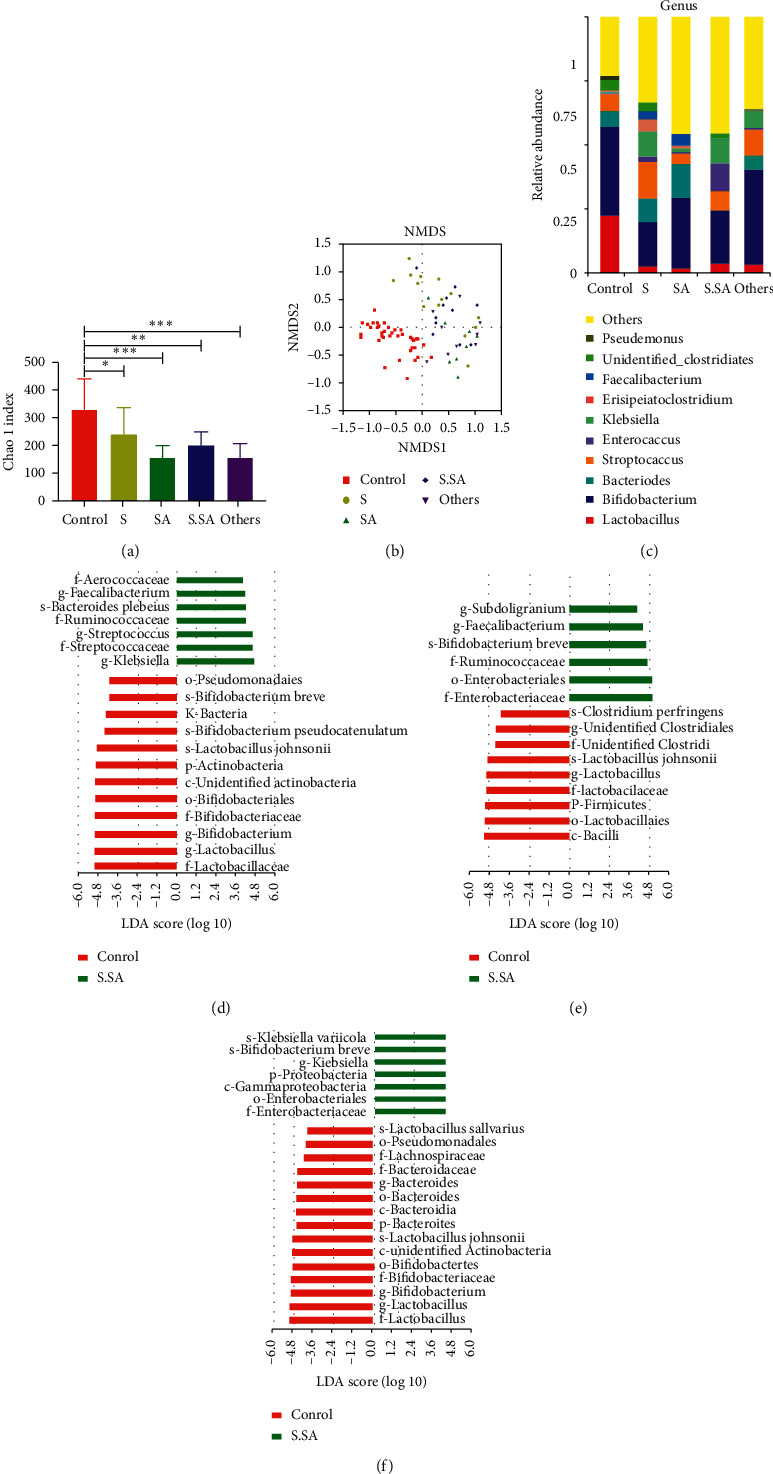
The gut microbiota of diarrheic infants infected by different pathogens. (a). The comparison of the alpha diversity (Chao 1 index) of different groups (control, *Salmonella* (S), *Staphylococcus aureus* (SA), combined *Salmonella* and *Staphylococcus aureus* (S.SA), and others) based on the OTU profiles. (b). The NMDS of beta diversity was calculated on ranking order. (c). The relative abundance of the top 10 microbiota at the genus level of different groups. (d–f). The LEFSe method analysis of the predominant bacteria between different groups.

According to Chinese health authorities, *Salmonella* and *Staphylococcus aureus* (SA) were the two common causes of infantile diarrhea in China (http://www.phsciencedata.cn/Share/zh-CN/index.jsp). Therefore, we identified the pathogens in the fecal samples of diarrheic infants and divided the samples by pathogen type: *Salmonella* (S), *Staphylococcus aureus* (SA), combined *Salmonella* and *Staphylococcus aureus* (S.SA), and others (no *Salmonella* or *Staphylococcus aureus*). Subsequently, we investigated the characteristics of the intestinal microbiota of diarrheic infants in the four groups. Regardless of the group, the Chao 1 index of diarrheic infants was reduced compared with healthy infants (Figure 6(a)). NMDS clustered the samples of infants with diarrhea into five groups; the *Salmonella* and *Staphylococcus aureus* groups were separated for healthy infants but did not separate from each other (Figure 6(b)).

At the genus level, the relative abundance of *Lactobacillus* and *Bifidobacterium* in the *Salmonella*, *Staphylococcus aureus*, combined *Salmonella*, and *Staphylococcus aureus* group was found significantly lower than in healthy infants (*P*=0.001). In contrast, the *Bacteroides* and *Streptococcus* in diarrheic infants were more abundant (*P*=0.001). Each group had different intestinal microbiota characteristics. For instance, *Bifidobacterium* and *Streptococcus* were predominant in the *Staphylococcus aureus* group, while the *Bifidobacterium* and *Bacteroides* account for a large proportion of bacteria in the *Staphylococcus aureus* group (Figure 6(c)). LEfSe analysis showed that, at the family level, the Ruminococcaceae and Enterobacteriaceae were differentially enriched in the *Salmonella* group (Figure 6(d)). In addition, at the family level, the most differentially abundant bacteria in healthy infants included Lactobacillaceae and Bifidobacteriaceae, while Streptococcaceae and Ruminococcaceae were overrepresented in the *Staphylococcus aureus* group (Figure 6(e)). At the genus level, *Klebsiella* and *Streptococcus* predominated in the *Staphylococcus aureus* group, while *Faecalibacterium* and *Subdoligranulum* were the predominant bacteria in the *Salmonella* group (Figure 6(f)).

There were changes in the gut microbiota of the infants infected with different pathogens compared with healthy infants. There were also differences in the gut microbiota between diarrheic infants infected by different pathogens. For instance, at the genus level (Figure 6(c)), the relative abundance of *Streptococcus* (14.93%) in the *Staphylococcus aureus* group was higher than in the *Salmonella* group (*P*=0.017) and the other group (*P*=0.047). Meanwhile, the relative abundance of *Enterococcus* (1.62%) in the *Staphylococcus aureus* group was higher than in the *Salmonella* group (*P*=0.023). The relative abundance of *Klebsiella* (10.45%) in the combined *Salmonella* and *Staphylococcus aureus* group was higher than in the *Salmonella* group (*P*=0.039).

## 4. Discussion

The homeostasis of the human gut microbiota has multiple positive effects on the host's health [23]. Microbes colonize the neonatal gut immediately following birth. The establishment and interactive development of the early gut microbiota play a vital role in infants' growth and health [24]. However, during the same period, owing to immune immaturity, the risk of illness will be high [25, 26]; for example, diarrhea often occurs.

Acute diarrhea was a diarrheal episode of presumed infectious etiology that begins quickly and lasts for fewer than 14 days [27]. Bacterial infections are a common cause of infantile diarrhea. Several different enteropathogenic agents can cause diarrhea in infants, such as enteroaggregative *Escherichia coli*, enteropathogenic *Escherichia coli*, *Salmonella*, *Shigella* spp., and *Staphylococcus aureus*, to name a few [28, 29].

Our study compared the characteristic of gut microbiota in healthy infants and diarrheic infants in multiple aspects. The results showed that the gut microbiota of infants with diarrhea changed significantly. First, compared with the healthy infants, the intestinal microbiota diversity of diarrheic infants was significantly decreased. Some research revealed that intestinal infection could affect the aerobic bacteria because they would spread through oxygen to obtain energy and metabolism [30–32]. Second, the Firmicutes accounted for a large proportion of bacteria in healthy infants and were beneficial to intestinal epithelial cells [33, 34]. However, in the diarrhea group, Proteobacteria was the predominant bacteria and the microbial signature of dysbiosis in gut microbiota. Third, our results showed that the relative abundance of *Lactobacillus* in the healthy infants was significantly higher than that in the diarrheic infants. On the other hand, Enterobacteriaceae had an opposite trend. Recent studies have found that intestinal dysbacteriosis was the leading cause of infantile diarrhea. Lactobacillaceae could alleviate the severity of diarrhea, whereas Enterobacteriaceae had an opposite effect [35]; our results are consistent with this finding.

Finally, when we compare the results of two groups at the genus level, we can find that *Lactobacillus* decreased and *Klebsiella* and *Enterobacter* increased with diarrhea. *Lactobacillus* are recognized as probiotics because of their health-promoting effects [36]. *Lactobacillus* can, via competitive exclusion, enhance epithelial barrier function and produce antipathogenic compounds to protect the host [37]. Davoodabadi et al. studied different *Lactobacillus* strains to identify probiotic candidates for preventing intestinal infections caused by diarrheagenic *E. coli* [38]. Szajewska et al. found that probiotic *Lactobacillus* could significantly reduce the risk of antibiotic-associated diarrhea in children and adults [39, 40]. Numerous bacterial infectious agents have been implicated in AAD, including *Clostridium perfringens, Staphylococcus aureus,* and *Klebsiella oxytoca* [41]*. K. oxytoca* also causes infections of the respiratory and urinary tracts and soft-tissue and hepatobiliary infections [42].

We also explored the effects of different ages on the gut microbiota of infants with diarrhea. The results showed no significant differences in species diversity among diarrheic infants at different ages. Meanwhile, beta diversity results suggested similar intestinal microbiota in infants with diarrhea at different ages. Interestingly, when we compared the effect of age on the relative abundance of bacterial species, we found differences between the lower and upper age groups. *Bifidobacterium*'s relative abundance was low in the gut microbiota of diarrheic infants of 0–120 days; cesarean delivery may be a factor. Studies have reported that cesarean-section infants show reduced intestinal microbiota complexity and relatively low abundance of *Bifidobacterium* [43, 44].

Meanwhile, the relative abundance of *Bifidobacterium* increased significantly with age, possibly because the infants had been breastfed since birth. Some studies reported high levels of *Bifidobacterium* and *Lactobacillus* in the fecal samples of breastfed infants [45, 46]. Similarly, we found that, in healthy infants, the relative abundance of *Lactobacillus* was low in the gut microbiota of diarrhea infants from 120 to 180 days, while *Bacteroides* increased. The reason may be that a newborn's intestine is aerobic, and only facultative anaerobic bacteria can grow. However, in just a few days, the intestinal cavity becomes anaerobic so that only *Bifidobacterium*, *Clostridium,* and *Bacteroides* can colonize [47]. In the first few weeks, the baby's intestinal microbiota resembles the mother's skin and vaginal microbiome, where *Enterococci*, Streptococcaceae, Lactobacillaceae, *Clostridium,* and *Bifidobacterium* predominate. In the first few months, the baby's diet is almost entirely milk, which is conducive to *Bifidobacterium,* the predominant microbiota at this stage [48, 49]. When solid food is introduced, the baby's gut microbiota will undergo a substantial change because the food contains various polysaccharides that are not easily digestible; as a result, the abundance of *Bacteroides* and *Clostridium* increases and *Bifidobacterium* and Enterobacteriaceae decrease [50, 51]. Therefore, it suggested that the changes in the gut microbiota of infants with diarrhea are related to the environment, feeding methods, and delivery methods and are not closely related to age from zero to one year.

Subsequently, based on PCR and sequencing results, we divided diarrheic infants into four groups: *Salmonella*, *Staphylococcus aureus*, *Salmonella,* and *Staphylococcus aureus* and others. The results of the microbiota analysis showed some differences between the groups. *Klebsiella* and *Staphylococcus* were the predominant bacteria in the *Staphylococcus aureus* group; meanwhile, Enterobacteriaceae was the most abundant bacteria in the *Salmonella* group. Therefore, diarrhea may be caused by mixed pathogenic bacterial infections. *Staphylococcus aureus* can secrete staphylococcal enterotoxin A (SEA) [52], which bind to MHC class II molecules and *T*-cell receptors to stimulate *T*-cell proliferation and activation by the variable region of *β* chain, leading to the uncontrolled increase of many proinflammatory cytokines. The superantigen SEA can cause fever, decrease immunity, and promote many other bacterial infections [53, 54]. Moreover, *Staphylococcus aureus*'s peptidoglycan can promote the occurrence of diarrhea by activating mast cells to release inflammatory substances [55]. Studies have reported reduced *Bacteroides* and increased *Klebsiella* in patients with *Staphylococcus aureus* infection [56, 57]; such finding is consistent with the results of this study. It is also reported that the use of antibiotics significantly increased the proportion of *Klebsiella* in intestine [58]. Hence, the cocolonization of *Staphylococcus aureus* and *Klebsiella* may be related to antibiotic exposure in the intestinal microenvironment. The relative abundance of Enterobacteriaceae increased, while the relative abundance of *Lactobacillus* decreased in the *Salmonella*-infected diarrheic infants; this trend is consistent with the previous finding on the characteristics of intestinal microbiota after *Salmonella* infection [59–61].

Similarly, patients receiving antibiotics also showed an increase in Enterobacteriaceae [62]. One might wonder why the *Salmonella* in diarrhea infants promotes Enterobacteriaceae. One possibility is that, after *Salmonella* infection, the pathogenic factors of *Salmonella* will be released to induce the host to develop a mucosal inflammation response [63]. As the host tries to eliminate the bacteria, it may cause “collateral damage” that destroys the human intestinal microecological balance, resulting in clinical symptoms like diarrhea.

In this study, we used high-throughput sequencing to investigate gut microbiota's characteristics in infants with diarrhea. We also compared the effects of different ages and different pathogens on the gut microbiota of diarrheic infants. The results showed that the gut microbiota of infants with diarrhea had changed significantly. Simultaneously, different pathogenic infections were found to affect the characteristics of gut microbiota in diarrheic infants; however, the intestinal microbiota of these infants at different ages was similar. This study was our first comprehensive analysis of the effects of different ages and different pathogen types on the gut microbiota in infants with diarrhea. It will provide some reference for the treatment of and nutritional adjustment for diarrheic infants. Indeed, the study's small sample size is a limiting factor; more samples are needed to verify our findings. Also, some infants were treated for skin problems, mostly eczema, followed by urticaria. Since rash in children is closely related to intestinal microbiota disorders and helper *T* cell imbalance [64, 65], the rash's underlying mechanism requires further study.

## Figures and Tables

**Table 1:** The basic clinical information on healthy infants and infants with diarrhea.

Characteristic		Healthy controls	Diarrheic infants
Number of samples	—	37	42
Gender	Male	24 (64.9%)	22 (52.4%)
	Female	13 (35.1%)	20 (47.6%)

Age	0–120 (days)	20 (54.1%)	8 (19.0%)
	120–180 (days)	17 (45.9%)	7 (16.7%)
	180–270 (days)	0	8 (19.0%)
	270–360 (days)	0	19 (45.2%)

Pathogen	S	0	14 (32.3%)
	SA	0	8 (19.0%)
	S.SA	0	11 (26.2%)
	Others	0	9 (21.4%)

Rash	—	0	26 (61.9%)

## Data Availability

All the data that were used to support the findings of this study are included within the article.

## Supplementary Materials

Supplementary Materials Identification of *Salmonella* infection in infants with diarrhea by agarose gel electrophoresis. PCR product was 284 bp.
