# Supplementary figures and images for: The Impact of Age and Pathogens Type on the Gut Microbiota in Infants with Diarrhea in Dalian, China
Source: Can J Infect Dis Med Microbiol. 2020 Nov 30;2020:8837156. doi: 10.1155/2020/8837156 (PMC7721492; doi:10.1155/2020/8837156)

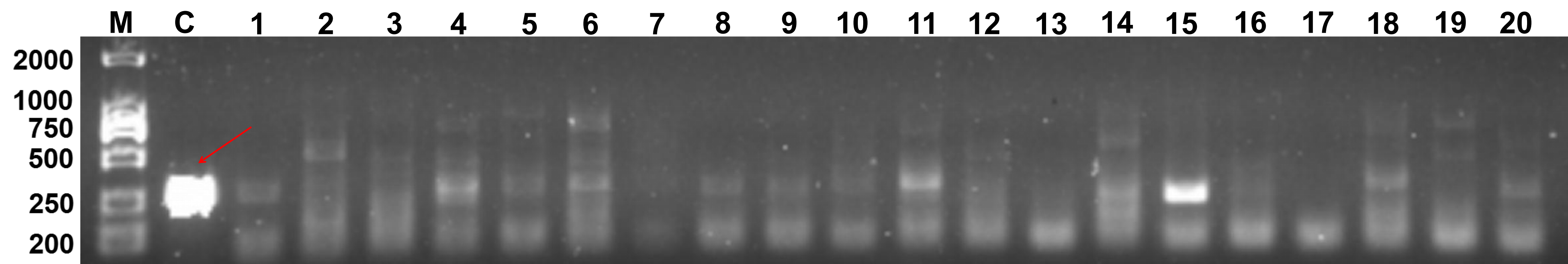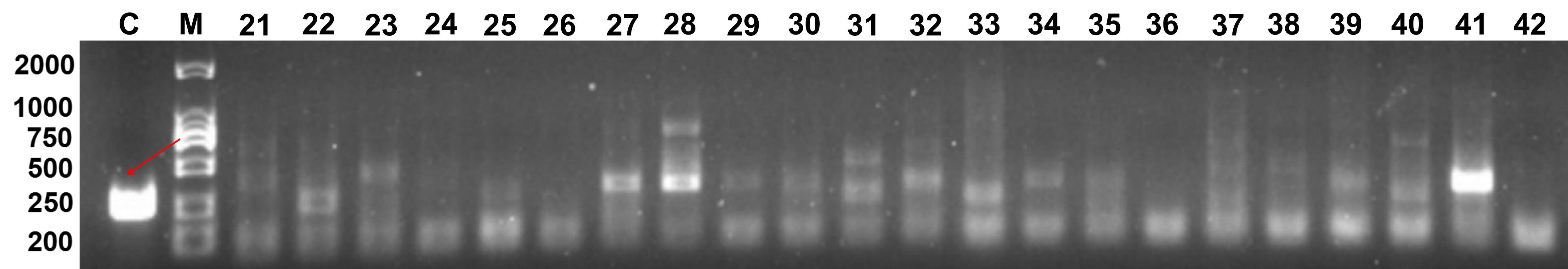

Supplement: Supplementary Materials — Identification of Salmonella infection in infants with diarrhea by agarose gel electrophoresis. PCR product was 284 bp. [file 8837156.f1.pdf]
